# The Transcriptional Landscape of Pericytes in Acute Ischemic Stroke

**DOI:** 10.1007/s12975-023-01169-x

**Published:** 2023-06-28

**Authors:** Carolina Buizza, Andreas Enström, Robert Carlsson, Gesine Paul

**Affiliations:** 1 Translational Neurology Group, Department of Clinical Science, Lund University, 22184 Lund, Sweden; 2 Department of Neurology, Scania University Hospital, 22185 Lund, Sweden; 3 Wallenberg Centre for Molecular Medicine, Lund University, 22184 Lund, Sweden

**Keywords:** Pericytes, Ischemic stroke, Single-cell RNA sequencing, Interleukin 11

## Abstract

The current treatment options for ischemic stroke aim to achieve reperfusion but are time critical. Novel therapeutic approaches that can be given beyond the limited time window of 3–4.5 h are still an unmet need to be addressed to improve stroke outcomes. The lack of oxygen and glucose in the area of ischemic injury initiates a pathological cascade leading to blood-brain barrier (BBB) breakdown, inflammation, and neuronal cell death, a process that may be intercepted to limit stroke progression. Pericytes located at the blood/brain interface are one of the first responders to hypoxia in stroke and therefore a potential target cell for early stroke interventions. Using single-cell RNA sequencing in a mouse model of permanent middle cerebral artery occlusion, we investigated the temporal differences in transcriptomic signatures in pericytes at 1, 12, and 24 h after stroke. Our results reveal a stroke-specific subcluster of pericytes that is present at 12 and 24 h and characterized by the upregulation of genes mainly related to cytokine signaling and immune response. This study identifies temporal transcriptional changes in the acute phase of ischemic stroke that reflect the early response of pericytes to the ischemic insult and its secondary consequences and may constitute potential future therapeutic targets.

**Supplementary Information:**

The online version contains supplementary material available at 10.1007/s12975-023-01169-x.

## Introduction

Stroke is the second leading cause of death worldwide and one of the main reasons behind adult disability [1].

In ischemic stroke, the interruption of nutrients and oxygen supply to the affected brain regions leads to a cascade of molecular and cellular events arising within seconds to minutes from the ischemic insult and progresses for days up to weeks. Stroke pathology develops in different phases that can be divided into a hyperacute, an acute, and a chronic phase [2]. The first few hours following the initial event correspond to the hyperacute phase, characterized by cell death, and breakdown of the blood-brain-barrier (BBB) [3, 4], followed by the acute phase which is usually considered to last for a few days, characterized by microglial and astrocytic activation, increased BBB permeability, vascular leakage, and inflammation [5]. The chronic phase starting days after the injury involve endogenous repair mechanisms including vascular remodeling, neural plasticity, and scar formation [6, 7].

Currently, available treatments, like tissue plasminogen activator (tPA) [8, 9] and thrombectomy, can only be given during a narrow time window within the first few hours after stroke onset, and are therefore available only to a small number of patients [8, 10]. As such, the development of therapeutic interventions that could target stroke pathogenesis beyond the hyperacute phase and improve stroke outcome is a global health priority.

Pericytes are one of the first cell types to respond to hypoxia [11, 12]. Surrounding the endothelial cell layer at the microvasculature, they form a key component of the BBB [13] and maintain BBB integrity and the vascular tone [14]. The response of pericytes to hypoxia may result in detrimental effects, e.g., early cell death contributing to restricted capillary blood-flow [15, 16] and their detachment from the capillaries that in turn leads to BBB breakdown [17]. However, pericytes also mediate beneficial effects in ischemic stroke, such as contributing to angiogenesis and stabilization of newly formed vessels [18, 19]. Pericytes may exert these different functions at different timepoints after stroke; however, the exact timeline and sequence of pericytes response associated with pathological events after ischemic stroke as well as the underlying molecular mechanisms governing this sequential response are currently not known [20]. Overall, their unique position at the blood/brain interface, their essential functions related to angiogenesis, and their ability to react to hypoxia potentially place pericytes at the top of the stroke cascade and make them an ideal candidate for therapeutic interventions that could regulate several key pathological hallmarks [21, 22].

One of the adaptive responses of pericytes to hypoxic environments is the expression of regulator of G-protein signaling 5 (RGS5), a negative regulator of G-protein-coupled receptors that in the brain is exclusively expressed by vascular mural cells, and a valuable tool to specifically identify pericytes in the brain [23–25]. Here, we utilize RGS5 reporter mice [26] in order to characterize the specific molecular pericyte response and potential differences between subpopulations of pericytes within the vascular niche in ischemic stroke at different timepoints of the acute ischemic cascade. To our knowledge, we present the first study comparing specific transcriptomic changes at a single-cell level between the ipsilateral and the non-affected contralateral hemispheres of the brain at 1, 12, and 24 h after the ischemic stroke insult. Overall, this study suggests a role of a subcluster of pericytes in ischemic stroke that warrants future investigation as a potential target population to intersect the pathological progression in stroke evolution.

## Materials and Methods

### Animals

We utilized C57bl/6 wildtype (WT) mice (*n*=10) and male RGS5^GFP/+^ mice (*n*=27) aged 8–12 weeks from a KO/knock-in reporter mouse strain that expresses green fluorescent protein (GFP) under the promoter of RGS5 in a C57bl/6 background [26]. In this model, one allele of RGS5 is replaced by GFP, making it possible to track pericytes by GFP expression under the activated RGS5 promoter.

RGS5^GFP/+^ mice were used for single-cell RNA sequencing (*n*=3), and real-time quantitative PCR (RT qPCR) verifications (*n*=24), of which *n*=12 were selected for pMCAO and *n*=12 for sham operations. Sham animals were used in order to further validate the use of the contralateral hemisphere as healthy control. Three WT mice for each RT-qPCR replicate were used as a negative control for GFP staining at the flow cytometer. *n*=7 additional WT mice underwent pMCAO for protein verifications by immunohistochemistry.

All animal experiments were approved by the Ethical Committee of Lund University (14205-199), and methods were carried out in accordance with the relevant guidelines and regulations. Animals were housed under standard conditions with a 12-h light/dark cycle and had access to food and water ad libitum. Every effort was made to keep animal numbers minimal according to the 3 R guidelines and principles of the Swedish Research Council (https://djurforsok.info/).

### Permanent Middle Cerebral Arterial Occlusion

In order to obtain a localized and reproducible stroke lesion, we used a pMCAO model. The distal part of the left MCA was occluded using electrocoagulation to induce focal ischemia as previously described [27]. Briefly, animals were anesthetized using 5% isoflurane initially, then 1.5–2% isoflurane was maintained during surgery. A total of 100 μl Marcain (2 mg/kg) (AstraZeneca) was locally applied to the site of surgery and a ~1-cm long incision was made between the left lateral ear and eye. The temporal muscle was detached from the skull in its apical and dorsal parts and the parotid gland moved aside. After identifying the MCA in the rostral part of the temporal area, dorsal to the retro-orbital sinus, a small craniotomy was made at the anterior distal branch of the MCA, using a surgical drill. After exposing the MCA, the artery was occluded using electrocoagulation forceps (ICC50; Erbe), proximal and distal to the bifurcation branch point. In the case of anatomical variation where no bifurcation was present, the MCA was coagulated twice, before the wound was sutured. Sham operations were conducted in the same way, but without occluding the MCA.

### Tissue Processing

At the respective timepoint, mice were transcardially perfused with 0.9% saline for 5 min. Brains for immunohistochemistry were extracted and frozen at −80 °C, then cut on a cryostat to coronal 20-μm-thick sections on glass slides. Sections were kept at −20 °C until further analysis. Brains for fluorescence-activated cell sorting (FACS) and single-cell sequencing were kept on ice and processed as described below.

### Tissue Dissociation for 10× Genomics

Bulk live non-neuronal cells were isolated according to Chang et al. [28]. After saline perfusion, the brains stroke ipsilateral cortical region or corresponding contralateral cortical region were minced with a razor blade and dissociated with enzymatic digestion using collagenase IV (400 U/ml, Worthington Biochemical, cat. LS004188), dispase I (1.2 U/ml, Worthington Biochemical, cat. LS02104), and DNAse I (32 U/ml, Thermo Fisher Scientific, cat. 18047019) in 6 ml phosphate buffer saline (PBS) with 0.9mM CaCl_2_ and 0.49 mM MgCl_2_ per sample. The solution was incubated at 37°C for 1 h with mechanical trituration every 10 min with 1000 μl followed by 100 μl pipette tips. After the tissue was disaggregated, 1 ml of fetal bovine serum (FBS) was added to the solution to stop the enzymatic reaction, filtered through a 40-μm filter, and centrifuged at 400 g for 5 min at 4 °C. The cells were washed once with ice-cold PBS before being centrifuged again at 400 × g for 5 min at 4 °C and resuspended in 10 ml of ice-cold 20% BSA in 1x PBS and centrifuged at 1000 × g for 25 min at 4 °C for removal of myelin and neurons. The cells were then resuspended in FACS buffer (0.5% BSA in PBS) and kept on ice for subsequent staining.

### FACS

For 10× genomics, single-cell suspensions were incubated with Fc-block for 15 min, followed by incubation with specific antibodies or corresponding isotype-matched control antibodies at a concentration of 1 μg/μl at 4 °C for 30 min in darkness in FACS buffer. To specifically isolate pericytes, we used RGS5-reporter mice (see methods). We verified the presence of live single-cell pericytes (CD140b^+^, CD13^+^, GFP^+^, DAPI^-^, PECAM1^-^) dissociated from endothelial cells among the processed cells before each experiment. We then sorted neuron depleted (see above) live DAPI^-^ bulk brain cells on ARIA-II. After sorting, a fraction of the cells was re-stained as explained above and re-analyzed in the flow cytometer to check for the survival of pericytes and endothelial cells (GFP^-^, PECAM1^+^, DAPI^-^) after sorting. Bulk-sorted cells were evaluated for > 80% viability with trypan blue staining. For each replicate of 10× analysis, 8500 cells were resuspended in 45 μl FACS buffer.

For qPCR-validation experiments of the scRNAseq data, single-cell suspensions were incubated with primary antibodies or corresponding isotype-matched control antibodies at 1 μg/μl at 4 °C for 30 min in darkness, after incubation with Fc-block as described above. Then, the cell suspensions were washed twice with PBS. For FACS sorting, quadrant gates were drawn to separate pericytes from the rest of the populations based on differences in specific antigen expression: pericytes were selected based on GFP^+^, CD140b^+^, CD13^+^, and PECAM1^-^, and all other cell populations were selected based on GFP^-^ (indicated as bulk), in order to compare the relative gene expression (Table 1). Cells were sorted on a FACS-ARIA II. To obtain a sufficient number of pericytes for subsequent RNA isolation, *n*=4 mice were pooled for each condition. For each replicate, one WT mouse was used as a negative control for GFP staining.

**Table 1:** FACS antibodies

Target	Supplier	Clone	Fluorophore
CD140b	eBioscience	APB5	APC
CD13	BD Pharmingen	R3-242	PE
PECAM1	Invitrogen	390	PE-Cy5
CD16/32	Biolegend	93	/

### Single-Cell Sequencing and Analysis

10× reactions were prepared with the v3.1 3’ x10 kit. cDNA and libraries were amplified according to the manufacturer’s (10× genomics) instructions. The single-cell libraries were sequenced on Illumina NOVA-seq (1- and 12-h samples) or HiSeq-seq (24-h samples) sequencers. The data was demultiplexed and genes were counted with the Cell ranger software (v.5). Cellranger aggr was run in order to normalize for sequencing depth between experiments. The Seurat suite v.4 was used to analyze and normalize the scRNA-seq data. Cells with a number of features <200 and a mitochondrial content above 10% were filtered out. The data was then log-normalized, and clusters were defined according to the highest number of clusters from the find-neighbors, find-clusters function in Seurat. Uniform manifold approximation and projection (UMAP) was run with the same number of dimensions not to under- or over cluster the data. To define cluster identities, we compared the expression of cell type-specific transcripts reported in the literature [29–36]. Differentially regulated genes (DEGs) were identified with the findallmarker- or findconservedmarker- functions in Seurat. Fast gene set enrichment analysis (FGSEA) was performed on the DEGs to define Hallmark gene set enrichments within the data [37, 38].

Matrix (M) and transport (T) pericytes were defined according to the gene list in [39]. By using the R package Ucell [40], the signatures for M- or T-pericytes were added to the Seurat object containing the mural cells in Fig. 2 from our dataset. The following mouse RNA transcripts were used to identify the two different populations. T-pericyte: *Slc20a2*, *Slc6a1*, *Slc1a3*, *Slc12a7*, *Slc6a12*, *Slc6a13*; M-pericyte: *Col4a1*, *Col4a2*, *Col4a3*, *Col4a4*, *Lama4*, *Adamts1*.

R Velocyto analysis [41] was performed on a .loom file generated from the 12-h ipsilateral stroke scRNAseq data. Endothelial cells (*Pecam1*^*+*^) and mural cells (*Pdgfrβ*^*+*^, *Rgs5*^*+*^, or *Acta2*^*+*^*,* respectively) were subclustered and trajectories were calculated for pseudotime nascient to mature RNA changes. *Il11* pericytes were identified as *Pdgfrβ*^*+*^, *Rgs5*^*+*^, *Il11*^*+*^, and *Acta2*^*-*^. The population that was on the way to becoming *Il11*-expressing pericytes was named “transient,” while the pericytes that were stationary in their nascent to mature RNA expression were denoted “stationary.” To identify what gene signatures differed between the transient and the stationary population, we isolated the individual cells with the “select cells” function in Seurat and R. Following selection, the DEGs between the transient and stationary pericytes were identified with the FindAllMarkers function in Seurat. Furthermore, a gene signature of the top 40 genes for transient pericytes was chosen for a Ucell signature add-on to the Seurat object. The transient top 40 gene signature was plotted across the 1-, 12-, and 24-h scRNAseq mural cell subclusters as UMAP.

### Real-Time Quantitative PCR

To further confirm single-cell data, we isolated pericytes as previously described from the ipsi- and contralateral hemispheres either from stroke mice or sham-treated mice at 24 h after the surgery. To ensure the collection of a sufficient number of sorted pericytes to perform the subsequent RT qPCR, cells from 4 mice were pooled for each condition, and the experiment was repeated 3 times. Total mRNA was isolated with RLTplus lysis buffer (Quiagen) from pericytes or bulk cells previously isolated by FACS. After mRNA purification, cDNA was retrotranscribed using Thermo Scientific Maxima First Strand cDNA Synthesis Kit for quantitative polymerase chain reaction (RT-qPCR). Samples were then prepared for qPCR using PowerUp™ SYBR® Green Master Mix (Thermo Fisher). Forward and reverse primers (TAG Copenhagen) of interest (Table 2) were added at a concentration of 0.05 μM/well. We then added 1 μl of cDNA from each sample and UltraPure water (Invitrogen) to reach the final volume of 10 μl/well. *Il11* master mix was supplemented with 0.5 M betaine (Sigma Aldrich) to enable PCR amplification of the GC-rich *Il11* transcript. The analyses were run on a Bio-Rad CFX96 RT qPCR system. Thermal cycling started with incubation at 50 °C for 2 min, followed by initial denaturation at 95 °C for 2 min. Cycling was made with 15 s denaturation followed by the annealing and extension at 60 °C for 1 min, for a total of 44 cycles. To calculate the relative target gene expression, the 2^−ΔΔCt^ method was used [42]. Each qPCR round of the triplicate was performed in duplicates measuring the relative expression of the genes from stroke ipsilateral pericytes in comparison with stroke contralateral pericytes.

**Table 2:** Primers

*Gene*	Forward primer	Reverse primer
*Adamts4*	CCCATTTCCCGCAGAACCAAG	CCTGCCGGGTGAACAGAATG
*Rdh10*	CAGGCATGGTTCGCCACATC	CCTCACCTTTTCCAGCTTGCAG
*Il6*	CGTGGAAATGAGAAAAGAGTTGTGC	GGAGAGCATTGGAAATTGGGGT
*Mt2*	TCTCGTCGATCTTCAACCGC	GCACTTGTCGGAAGCCTCTT
*Fstl1*	GAAGCCTCTGTGTTGACGCC	TCCTCCAGGGCACACTTCTTC
*Dbn1*	GTCGTCCGTACTGCCCTTTCA	AGTCTCCTGGGCCTCTTGAGT
*Ednrb*	CTGCGGAGGTGACCAAAGGA	TCCTCTGCGAGCAACTTGTAGG
*Stc1*	TCGCCAATGGGATCACCTCC	CGGACAAGTCTGTTGTAGTATCTGT
*Ccl2*	CTTCCTCCACCACCACCATGCAG	AAGGCATCACAGTCCGAGTCA
*β2m*	TTCTGGTGCTTGTCTCACTGA	CAGTATGTTCGGCTTCCCATTC

Samples with a normal *β2m* amplification curve but an amplification of the target gene above 36 qPCR cycles were considered undetected and imputed with the R package nondetects [43].

### Immunohistochemistry

Sections were rehydrated for 5 min with PBS and fixed in a solution of 4% paraformaldehyde (PFA) in PBS. Sections were then permeabilized with Triton-X 100 (Tx) 1% solution in PBS and incubated with a blocking solution (10% normal goat or donkey serum, 1% Tx-PBS) for at least 1 h at room temperature (RT), followed by incubation overnight at 4 °C with primary antibodies diluted in the blocking solution (Table 3). Platelet-derived growth factor receptor beta (PDGFRß) was used as pericyte marker. Sections were incubated with the corresponding secondary antibodies diluted in PBS for 1 h at RT and with DAPI (1:500) for 5 min (Table 4). Negative controls were treated similarly without primary antibodies. Sections were washed in PBS and rinsed in deionized water and mounted using PVA-DABCO.

**Table 3:** Primary antibodies

Target	Dilution	Clone	Supplier	Catalog number
IL11	1:40	188520 Rat	R&D Systems	MAB418
PDGFRß	1:100	G.290.3 Rabbit	Thermo Fisher	MA5-15143
CD13	1:100	VRR011509	R&D Systems	AF2335-SP

**Table 4:** Secondary antibodies

Target	Dilution	Clone	Supplier	Catalog number
Cy3	1:500	Donkey anti-rat	JacksonImmuno	712-165-153
Alexa 647	1:500	Donkey anti-rabbit	JacksonImmuno	711-605-152

### Image Processing

Immunofluorescence images were acquired on a Leica DMi8 confocal microscope using the ×40 oil immersion objective. The tissues were visually scanned by the operator in order to find IL11 signals and all the locations in which a signal was present were imaged, for a total of 70 images from 7 different animals. The images had *xy* dimensions of 387.69 × 387.69 μm and a depth of 6.5 μm and were acquired with a *z*-step size of 0.35 μm. Images were analyzed and assembled using ImageJ software v.1.53v (NIH, USA). 2D images were produced from the z-stack using Max Intensity function, followed by Split Channels function. The threshold for the IL11 channel was set using the Max Entropy automatic threshold, while for PDGFRβ channel, Moments threshold was set. A cut-off of the particles from the debris was set using Analyze Particles function (IL11 channel, 0.4-Infinity; PDGFRβ channel, 1.5-Infinity). Measurements of area density were conducted on the resulting images using the area fraction measurement tool of ImageJ. The area density was expressed as the percentage of IL11 and PDGFRβ of the total image area.

### Statistical Analysis

To analyze qPCR data, one-way ANOVA with a 95% confidence level was used, followed by Dunnett’s multiple comparison correction test. To analyze confocal images, paired *t*-test with a 95% confidence level was used. Statistical analysis was performed using GraphPad Prism version 9.0.0 for Mac, GraphPad Software, San Diego, CA, USA, www.graphpad.com.

### Data Availability

The data that support the findings of this study are openly available in Gene Expression Omnibus at https://www.ncbi.nlm.nih.gov/geo/, reference number GSE234052.

## Results

### ScRNA-seq Analysis Identifies Differences in Cell Populations in the Acute Phase After Ischemic Stroke

**Fig. 1:**
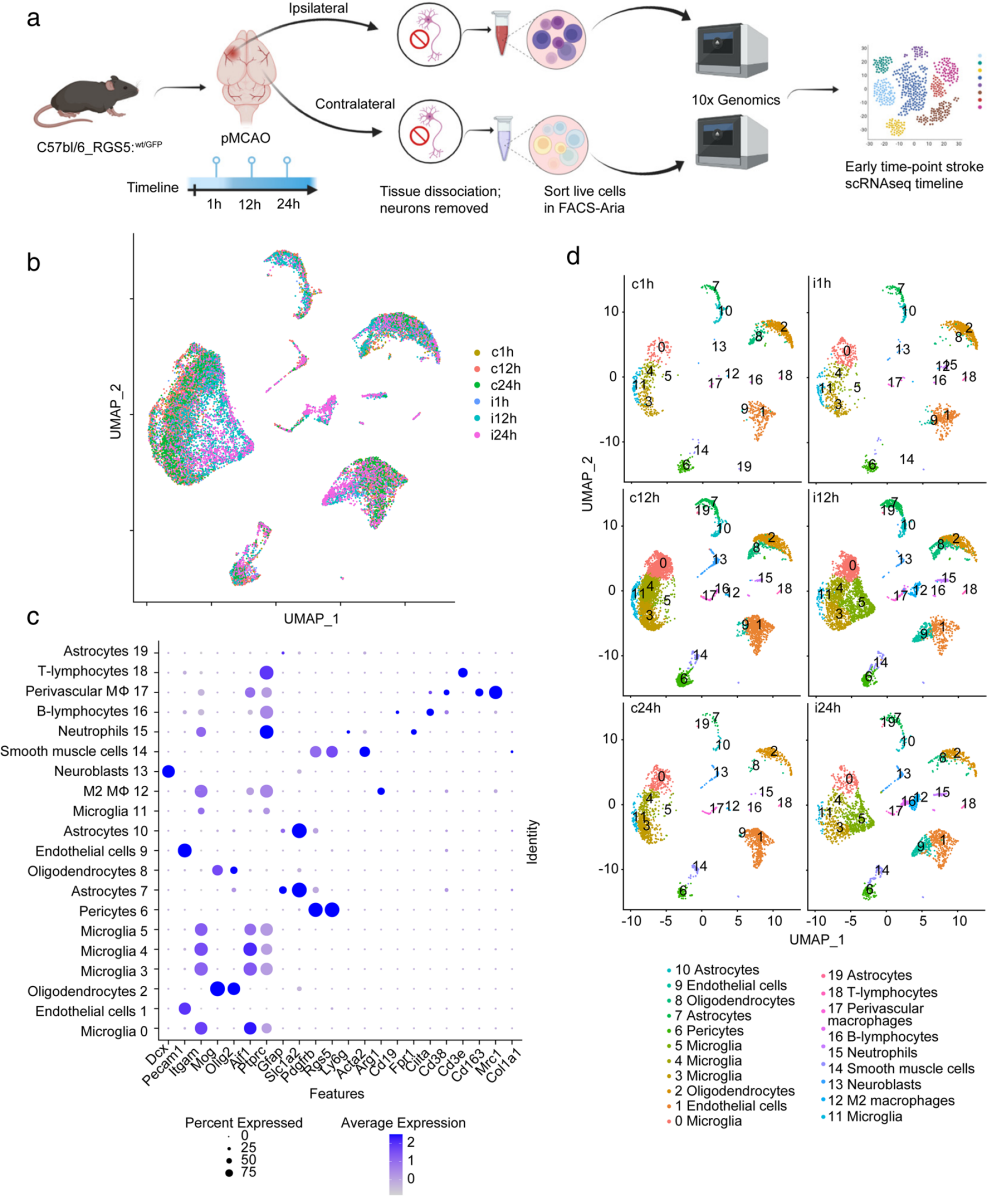
Single-cell sequencing of non-neuronal cells 1, 12, or 24 h after pMCAO stroke in mice. **a** Graphical diagram of the single-cell isolation and scRNA-seq experimental setup. **b** Visualization and clustering of the scRNA-seq data in Seurat from all the timepoints and areas analyzed in the study. **c** Cell types identified by clustering analysis and gene expression of the genes defining cell type identity. **d** UMAP plotted singularly for different timepoints and hemispheres. c contralateral; i ipsilateral; h hour

For the transcriptomic analysis, brain cells depleted of mature neurons were isolated from either the ipsilateral (ipsi, i) or the healthy contralateral (contra, c) hemispheres at 1, 12, or 24 h (Fig. 1a). From the sequencing, a total of 23,675 cells with a mean of 2,020 genes per cell were obtained. UMAP plots of the aggregated data from the different sequencing runs showed overlapping clusters, indicating successful integration of the data (Fig. 1b). Using known cell-type markers or the combination of unambiguous genes for certain cell types, we identified 19 different clusters, including microglia (*Itgam*^*+*^, *Aif1*^*+*^, *Ptprc*^*+*^); endothelial cells (*Pecam1*^*+*^); oligodendrocytes (*Mog*^*+*^, *Olig2*^*+*^); pericytes (*Pdgfrβ*^*+*^, *Rgs5*^*+*^, *Acta2*^*-*^); astrocytes (*Gfap*^*+*^, *Slc1a2*^*+*^); M2 macrophages (*Itgam*^*+*^, *Aif1*^*+*^, *Ptprc*^*+*^, *Arg1*^*+*^); neuroblasts (*Dcx*^*+*^); smooth muscle cells (SMC) (*Pdgfrβ*^*+*^, *Rgs5*^*+*^, *Acta2*^*+*^); and others (Fig. 1c, Suppl. Table 1). To gain more certainty regarding the identity of pericytes, we conducted an analysis of additional well-established markers, namely *Abcc9*, *Anpep*, *Cspg4*, and *Atp13a5*, recently suggested to be a specific indicator of pericytes in the brain [44] (Suppl. Fig. 1).

Major changes in cellular clustering between the ipsilateral and the contralateral hemispheres were detected 12 and 24 h after stroke (Fig. 1d); however, subtle changes were also detected at 1 h, including a reduction in the oligodendrocyte population density, passing from 7.3 to 1.6% of the total amount of cells, and the onset of a small cluster of neutrophils amounting to 0.85% of the total cells in the ipsilateral hemisphere compared to the contralateral one (Fig. 1d). At 12 h, we observed an increased density of cells in the microglial cluster 5 (from 1 to 18.9% of the total) and a decrease in the density of cells in the microglial cluster 4 (from 18.8 to 8.3%) in the ipsilateral hemisphere compared to the contralateral. At 24 h, the differences between the ipsilateral and contralateral hemispheres were more marked than at the 12-h timepoint, with an increased density of microglial cluster 5 (0.9 to 17.6%), endothelial cells cluster 9 (0.4 to 11.1%), macrophages (0.2 to 8.5%), and a decrease in the microglial clusters 0, 3, and 4 and in the endothelial cell cluster 1 (13.2 to 5.4%) (Fig. 1d; Suppl. Fig. 2).

### Temporal Heterogeneity of Pericyte Sub-populations in the Acute Phase of Ischemic Stroke

**Fig. 2:**
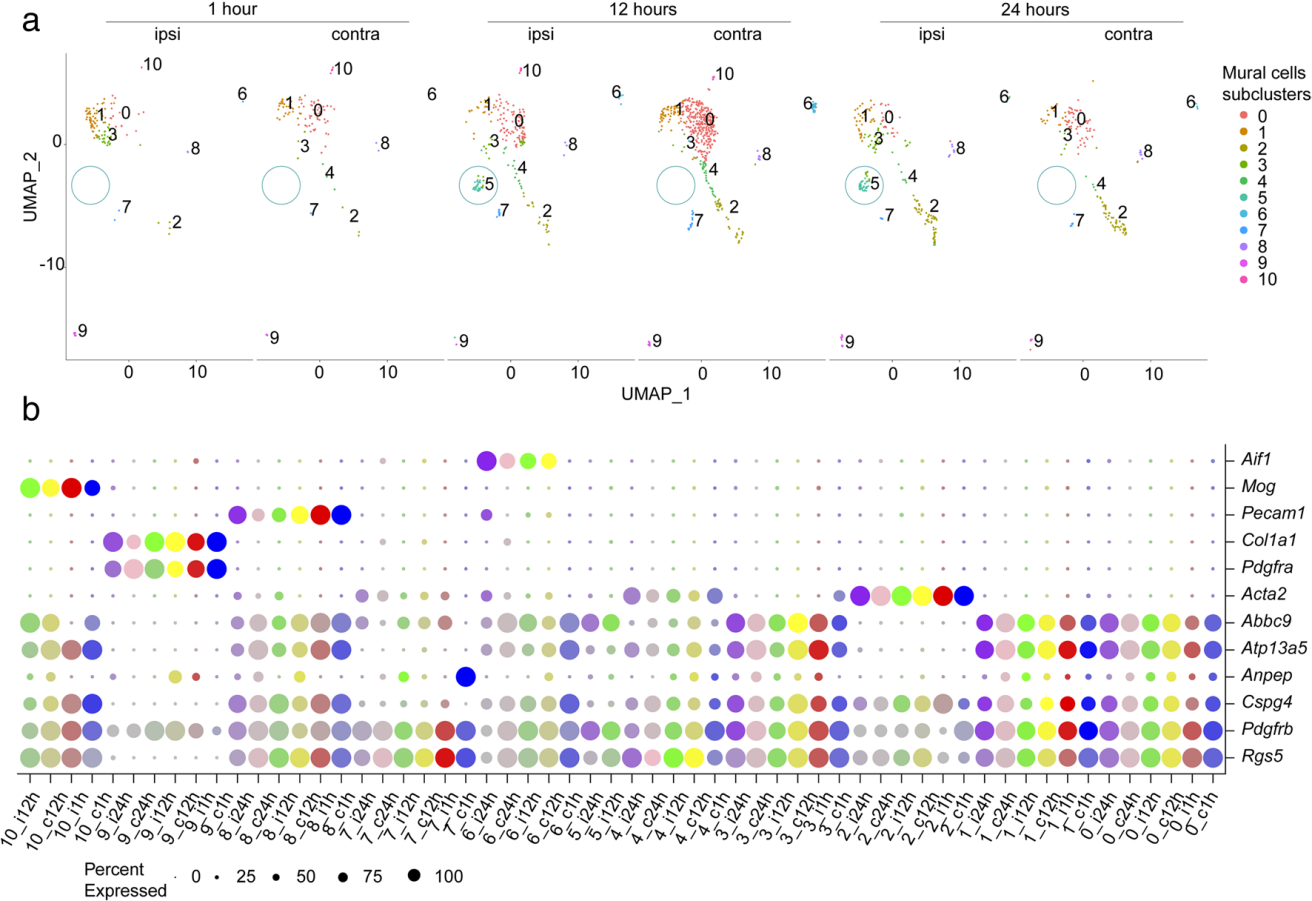
Heterogeneity of mural cells in the acute phase of ischemic stroke. **a** Re-clustering analysis on pericytes and smooth muscle cells reveals 8–10 different subclusters depending on the timepoint and experimental condition. The pericyte subcluster 5, stroke-specific, is highlighted. **b** Dot plot representing the marker expression of each mural cell subcluster. Different timepoints and hemispheres are denoted by colors. The size of the dots equals the percentage of the population expressing the marker, and the intensity of the color denotes the expression level, respectively. Pericytes are defined as *Pdgfrβ*^*+*^, *RGS5*^*+*^, *Atp13a5*^*+*^, *Abcc9*^*+*^, and *Acta2*^*-*^, SMCs *Acta2*^*+*^, fibroblasts *Pdgfrβ*^*+*^, *Pdgfrα*^*+*^. Clusters 6, 8, and 10 are pericytes co-expressing markers of pericytes and microglia (*Aif1*^*+*^), endothelial cells (*Pecam1*^*+*^), or oligodendrocytes (*Mog*^*+*^). c contralateral; i ipsilateral; h hour

To further investigate the specific pericyte transcriptomic response after ischemic stroke, we extracted the pericytes and SMC clusters (6 and 14, respectively) from the dataset in Fig. 1 and performed a new clustering analysis (Fig. 2). Pericytes are widely known as highly plastic cells and share common features with SMC; therefore, we decided to also include SMC in our re-clustering analysis to obtain a more comprehensive view of the temporal changes after stroke onset. The re-clustering analysis revealed 8–10 subclusters depending on the timepoint and experimental condition (Fig. 2a). Subclusters 0, 1, 3, and 5 were classified as pericytes (*Rgs5*^*+*^, *Pdgfrβ*^*+*^, *Atp13a5*^*+*^, *Abbc9*^*+*^, and *Acta2*^-^) (Suppl. Fig. 3a), subclusters 2, 4, and 7 as *SMC* (*Rgs5*^*+*^, *Pdgfrβ*^*+*^, *Acta2*^*+*^), subcluster 9 as fibroblasts (*Pdgfrα*^*+*^, *Pdgfrβ*^*+*^, *Col1a1*^*+*^). Subclusters 6 (partly *Pecam1*^*+*^), subcluster 8 (partly *Aif1*^*+*^), and subcluster 10 (partly *Mog*^*+*^) were classified as potential doublets of endothelial cells, microglia, and oligodendrocytes, respectively (Fig. 2b).

When comparing the ipsilateral and contralateral hemispheres 1 h after stroke, the frequency of cells in subcluster 3 increased, while we observed fewer cells in subcluster 0 (Fig. 2a; Suppl. Fig. 3b). Similarly, at 12 and 24 h after stroke, the cellular frequency in subcluster 3 increased while it decreased in subcluster 0 (Fig. 2a; Suppl. Fig. 3b). Interestingly, the pericyte subcluster 5 was only present in the ipsilateral sample at both, 12 and 24 h, but not in the contralateral hemisphere, suggesting a unique pericyte subpopulation in response to ischemic stroke (Fig. 2a; Suppl. Fig. 3b). Other subclusters that showed a different distribution across the timepoints were clusters 6, 8, and 10, all characterized by a low frequency of cells (Fig. 2a; Suppl. Fig. 3b).

The concept of pericyte subtypes is still an area of active research, and there are ongoing discussions and debates regarding their characterization and classification. Some studies have proposed the existence of distinct pericyte subtypes based on differential gene expression patterns, localization within blood vessels, or function. These subtypes have been tentatively labeled as type 1 and type 2 pericytes [45–47], A- and B-pericytes [48], or T- (transport) and M- (matrix) pericytes [39], but their specific characteristics may vary depending on the study. For this study, we compared the pericyte subclusters to the T- and M-pericytes as defined in [39] to understand if we could gather the cells under a specific function. Based on this analysis, the pericyte subcluster 5 seemed to belong to the M-pericytes (Suppl. Fig. 4a–b), suggesting a role in the extracellular matrix organization.

### DEGs and GSEA Analyses on the Most Responsive Pericyte Subclusters

To further explore the specific response of selected subclusters, we investigated the differentially expressed genes (DEGs) between the selected subclusters and the remaining pericyte clusters at each specific timepoint.

**Fig. 3:**
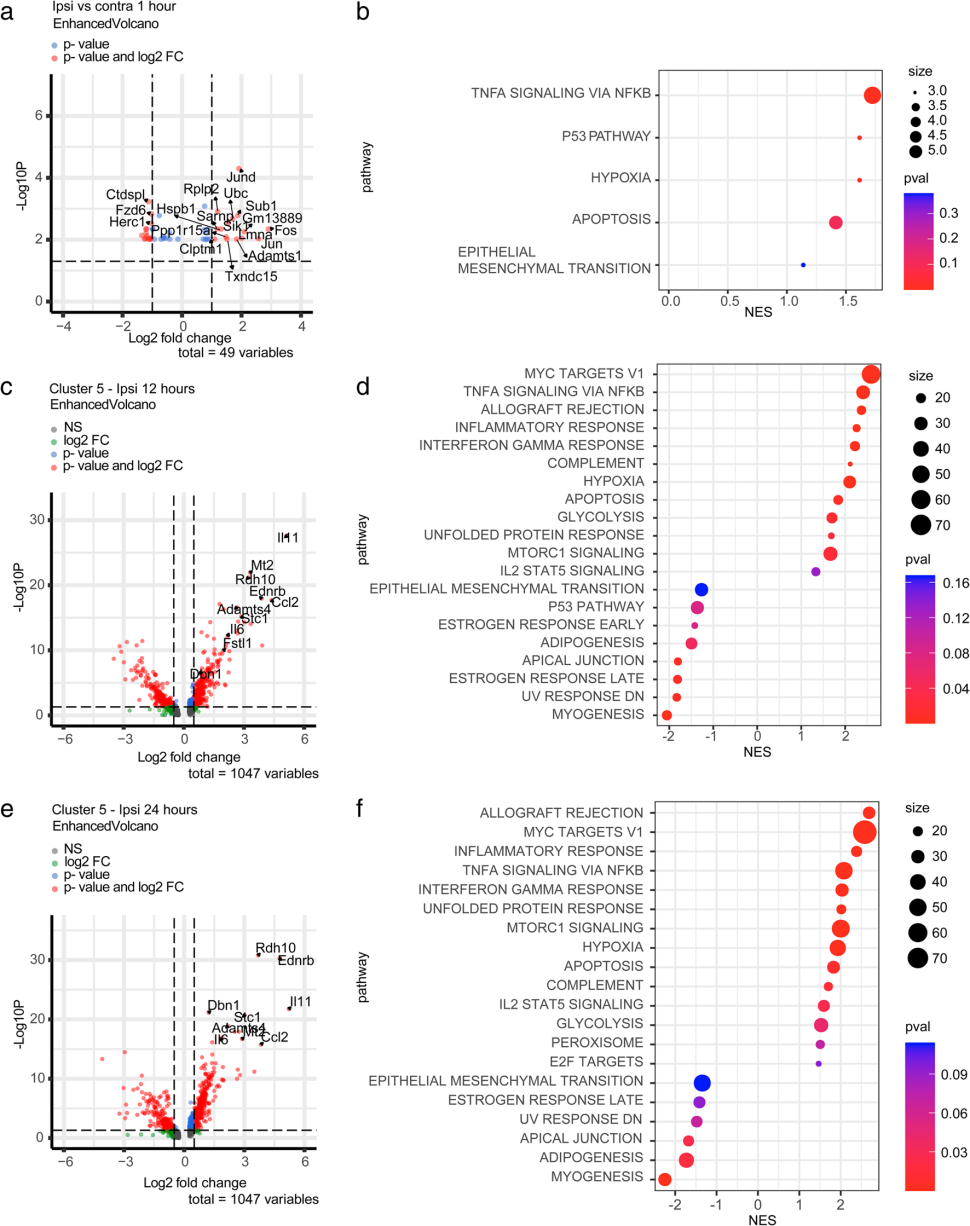
DEG analysis and GSEA on the mural cells subclusters. **a** Volcano plot displaying DEGs in the pericyte subcluster 3 1 h after ischemic stroke. **b** Hallmark gene sets from MSigDB on the differentially expressed genes from subcluster 3 between ipsilateral and contralateral hemispheres 1 h after stroke. **c** Volcano plot displaying the genes enriched in cluster 5 at 12 h after stroke. **d** Hallmark gene sets on the differentially expressed genes from cluster 5 compared to the other mural cell subclusters 12 h after stroke. **e** Volcano plot displaying the genes enriched in cluster 5 compared to the other mural cell subclusters at 24 h after stroke. **f** Hallmark gene sets on the differentially expressed genes from cluster 5 24 h after stroke. Cut-off DEG *p* value < 0.05; fold change > 0.5

At 1 h after stroke, DEG analysis on the pericyte-specific subclusters revealed that, in the ipsilateral hemisphere compared to the contralateral one, the most active subcluster of pericytes upregulated JunD proto-oncogene (*Jund*), Fos proto-oncogene (*Fos*), Jun proto-oncogene (*Jun*), SUB1 regulator of transcription (*Sub1*), SIK family kinase 3 (*Sik*), ADAM metallopeptidase with thrombospondin type 1 motif 1 (*Adamts1*), and others (Fig. 3a).

For the 12- and 24 h-timepoints, we focused on the DEGs between cluster 5 and the remaining mural cell subclusters (Fig. 3c and e), as it was the only cluster appearing selectively in the ipsilateral hemisphere after stroke, and therefore classified as stroke-specific. Based on the number of genes and the fold change of expression, the largest difference in DEGs was observed 24 h after stroke, where the 10 top most upregulated genes included the following: interleukin 11 (*Il11*), interleukin 6 (*Il6*), retinol dehydrogenase 10 (*Rdh10*), endothelin receptor B (*Ednrb*), C-C motif chemokine ligand 2 (*Ccl2*), metallothionein-2 (*Mt2*), stanniocalcin 1 (*Stc1*), ADAM metallopeptidase with thrombospondin type 1 motif 4 (*Adamts4*), drebrin 1 (*Dbn1*), and follistatin like 1 (*Fstl1*) (Fig. 3e). Upregulated DEGs were generally the same between 12 and 24 h.

To investigate the top pathways that could best explain the variations among the most interesting pericytes subclusters based on DEG analysis, we performed an unbiased search across the MSigDB “Hallmark” gene sets.

First, we evaluated the most frequently differentially expressed gene sets between the ipsilateral and contralateral pericytes clusters 1 h after stroke. Hallmark analysis showed an upregulation of TNFa signaling, P53 pathway, hypoxia, and apoptosis (Fig. 3b).

Secondly, to gain insights into the functions associated with the pericyte subcluster 5, we performed the GSEA on the DEGs of cluster 5 vs the rest of the mural cells subclusters. Twenty-four hours after stroke, cells from subcluster 5 upregulated pathways related to downstream Myc targets, tumor necrosis factor (Tnfα) via nuclear factor kappa-light-chain-enhancer of activated B cells (NF-κB), interleukin-2 (Il2)/signal transducer and activator of transcription 5 (Stat5), mammalian target of rapamycin complex 1 (Mtorc1), hypoxia, apoptosis, unfolded protein response, glycolysis, and pathways related to inflammation. Downregulated pathways included epithelial mesenchymal transition, estrogen response, UV response, apical junction, adipogenesis, and myogenesis. GSEA analysis on the markers of the stroke-specific cluster 5 at 12 h compared to the remaining mural cell subclusters from the same timepoint revealed a similar pathways profile to 12 h (Fig. 3d and f).

### Velocyto Predicts a Transient Pericyte Population Giving Rise to Subcluster 5

In order to study where the *Il11*-expressing subcluster of pericytes originates from in terms of transcriptomic origin and pseudo-time analysis, we employed Velocyto [41] to determine the difference between nascent and mature RNA transcripts in 12h IPSI pericyte and endothelial cell subclusters. Pericytes display a propensity to generate *Pgdfrb*^+^, *Rgs5*^+^, *Il11*^+^, and *Acta2*^-^ pericytes from a fraction of the pericytes (Suppl. Fig. 5a–d), as indicated from the trajectories (arrows) of these “transitional” pericytes compared to the more “stationary” pericytes (Suppl. Fig. 5e, f). To identify the difference between the transitional and the stationary population of pericytes, we isolated the gene expression of transient pericytes (Suppl. Fig. 5f) and compared it with the expression of the stationary pericyte subcluster (Suppl. Fig. 5e, g).

Then, we plotted the top 40 genes that distinguished transient from stationary pericytes in the UMAP from Fig. 2 (Suppl. Fig. 5h). The transitional top 40 genes were highly expressed in the *Il11-*expressing pericytes and in a fraction of the subcluster 3, both at 12 and 24 h, suggesting that the *Il11* pericytes are derived from subcluster 3, and that the top 40 gene-set expression is maintained in the more stable *Il11* subcluster pericytes.

### RT qPCR Validation of the Top 10 DEGs Associated with the Stroke-Specific Subcluster of Pericytes

Since the transcriptomic profile of the pericytes subcluster 5 showed the largest changes at 24 h after ischemic stroke, we decided to validate the increased gene expression of the DEGs characterizing the stroke-specific pericyte subcluster using RT-qPCR. Thus, we isolated pericytes by cell sorting using the same RGS5^GFP/+^ reporter mice and compared the expression of the genes selected from the transcriptomic analysis to the remaining cell types 24 h after stroke.

**Fig. 4:**
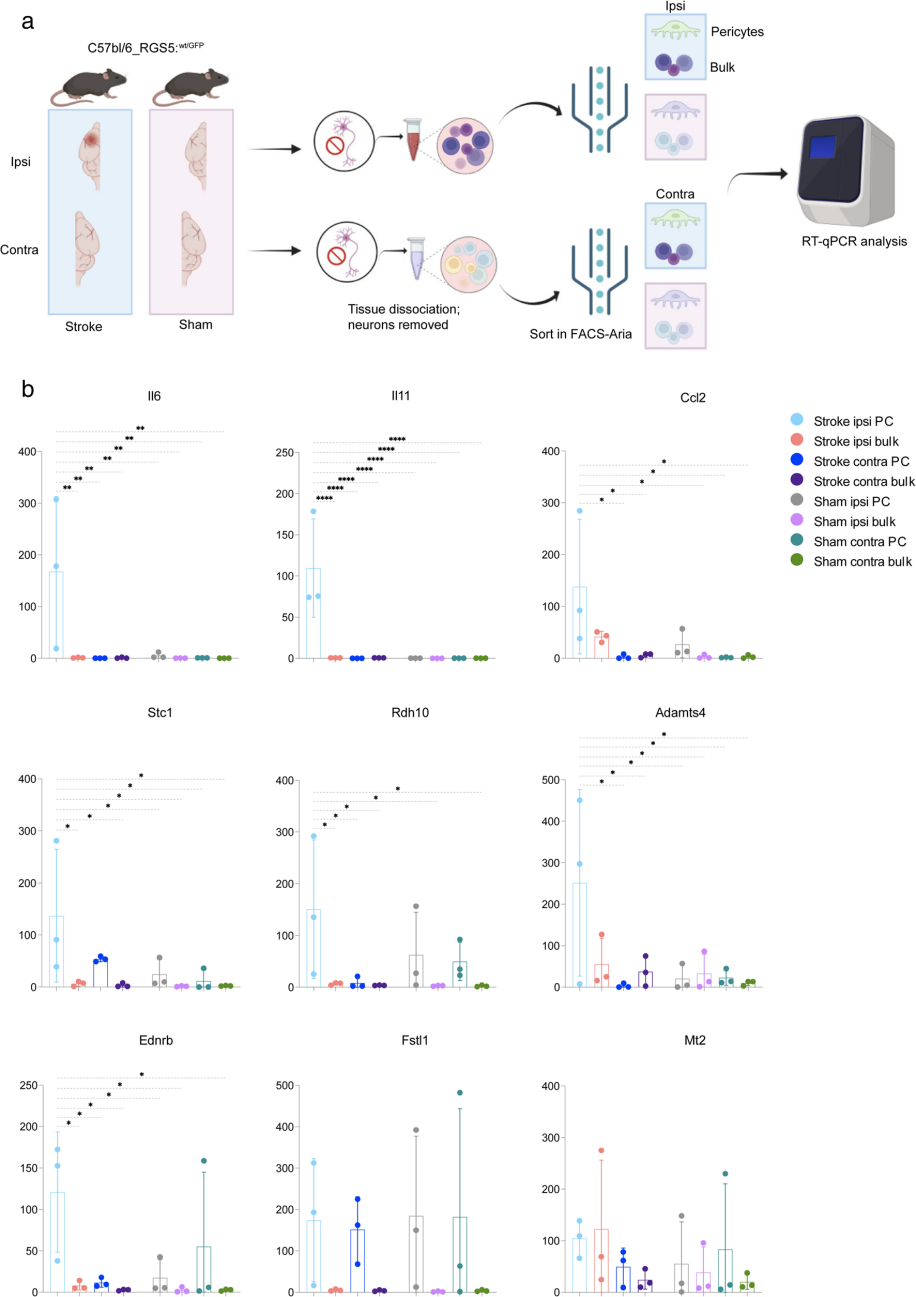
RT-qPCR validation of RNA sequencing data for the top 10 most differentially upregulated genes by the stroke-specific subcluster 5 of pericytes 24 h after stroke. **a** Graphical diagram of single-cell isolation and FACS sorting for RT-qPCR experiments. **b** Bar graphs show that mRNA values of *Ednrb*, *Rdh10*, *Stc1*, *Il6*, *Il11*, *Ccl2*, *Mt2*, *Adamts4*, and *Fstl1* presented as mean of experimental replicates ± SD (*n* = 4 pooled mice per replicate, 3 replicates). * = 0.05 > *P* > 0.001; ** = 0.01 > *P* > 0.001; *** = 0.001 > *P* > 0.0001; **** = *P* <0.001

Overall, qPCR analysis confirmed our sc-RNA seq data (Fig. 4b). *Il6*, *Il11*, and *Adamts4* mRNA expression by ipsilateral pericytes was respectively ~180, ~110, and ~120 times increased compared the other groups, where the mRNA expression was low or non-detectable. *Rdh10* and *Stc1* mRNA levels showed a ~150-fold change increase in the ipsilateral pericytes and only a slight upregulation of ~50 and ~20 in the contralateral pericytes. *Ccl2* mRNA levels were ~120 times upregulated in ipsilateral pericytes, although the remaining cells from the ipsilateral hemisphere also showed a ~20-fold change increase in *Ccl2* expression. *Ednrb* mRNA levels were mostly increased in ipsilateral pericytes with a ~120-fold change increase, despite one sample of contralateral pericytes (from the sham-treated animals) that also expressed *Ednrb* mRNA. qPCR results on *Fstl1* levels only partly confirmed the transcriptomic data, being upregulated in all groups of pericytes in stroke animals, both in the ipsilateral and the contralateral hemisphere, but not in the groups of bulk cells. *Mt2* mRNA was expressed by ipsilateral pericytes, but with a wide variation in the expression in the other groups, therefore not confirming the computational prediction on the differentially expressed genes from the transcriptomic analysis. Finally, *Dbn1* expression was too low to be accurately detected with qPCR analysis (data not shown).

### IL11 Is Exclusively Expressed in PDGFR-Expressing Cells After Stroke

**Fig. 5:**
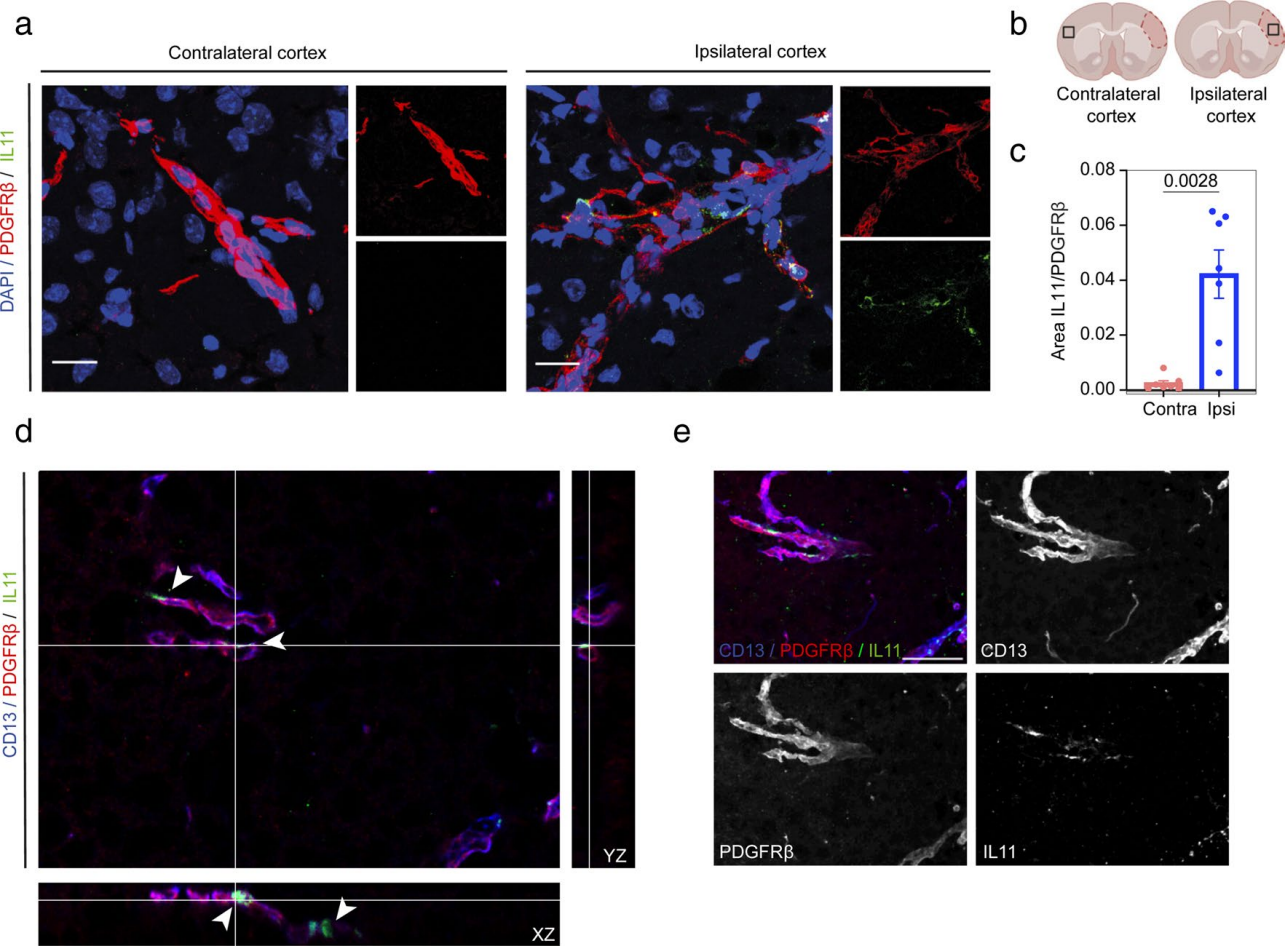
Validation of IL11 increased expression in the stroke ipsilateral hemisphere by immunohistochemistry. **a** IL11 is only detected in the ipsilateral hemisphere of mice subjected to stroke in the proximity of pericytes. **b** Location of the selected images. The infarct core is outlined. **c** Area fraction occupied by IL11 and PDGFR𝛽 signals. Data are presented as mean ± SEM. *P* = 0.028, paired *t*-test 95% confidence level (confidence intervals 0.01985 to 0.05979). Scale bar = 20 μm. **d** Orthogonal views showing IL11 expression in PDGFR𝛽 and CD13 expressing cells in the ipsilateral cortex and **e** 2D maximum projection of the merged channels. *z* stack = 24 μm; scale bar = 25 μm. Arrow points at IL11 signal

Interestingly, both bioinformatic analysis and qPCR indicated that *Il11* expression was among the genes with the highest fold change and specific to pericytes from subcluster 5 at 12 and 24 h. IL11 is a cytokine known to be primarily produced by stromal fibroblasts within the gastrointestinal tract [49], heart [50], liver [51], and lungs [52], but it has also been reported that epithelial and immune cells can be a source of IL11 under pathological conditions [53, 54]. Hence, we decided to further evaluate IL11 protein spatial expression in pMCAO brains using immunohistochemistry. Confocal imaging confirmed the significant increased expression of IL11 in the ipsilateral hemispheres (*P* = 0.028; CI, 0.01985–0.05979) and highlighted that IL11 presence was only restricted to pericytes (PDGFRβ and CD13 expressing cells) and not expressed in other cell types (Fig. 5a–e).

## Discussion

Extending previous studies by our group investigating functions of pericytes in ischemic stroke [55–58], we here perform a further characterization and an in-depth analysis of the molecular response of RGS5-expressing pericytes to ischemic stroke using the same stroke model, pMCAO. To explore the events that determine BBB breakdown and vascular disruption, we examined the transcriptomic signature of pericytes at three different timepoints after stroke and we identified several transcripts uniquely expressed in pericytes that change over time and may constitute potential targets for the treatment of early vascular dysfunctions associated with ischemic stroke. To our knowledge, this is the first study to focus specifically on the temporal dynamics of molecular mechanisms engaged by pericytes during the acute phase of the pMCAO model using single-cell transcriptomic analysis.

Our data show that pericytes in the ischemic stroke area upregulate *Il6* compared to the other cell types at both 12 and 24 h after stroke. In addition, our data also revealed that *Il11* expression is only upregulated in pericytes in the stroke-specific subcluster 5 but absent in the contralateral pericytes or in other cell types. IL6 and IL11 belong to the same cytokine family [59]. IL6 has been described to have a bidirectional role in ischemic stroke, being both harmful and protective [60], while IL11 seem to have a protective role after ischemic stroke [61], despite having controversial roles in other processes such as fibrosis and inflammation [27]. Our findings are consistent with previous studies, describing pericyte production of IL6 as early as 2 h after hypoxic treatment [21] and *Il11* expression 24 h after ischemic stroke [20, 62].

We validated the selective expression of IL11 protein in pericytes after stroke using immunohistochemistry, showing IL11 exclusively colocalizing with pericytes, and not with neurons or other cell types [61].

IL6 and IL11 act mainly on the JAK-STAT pathway [63]. Our group has previously shown that in hypoxia pericytes activate STAT3 pathways even before the widely studied hypoxia-inducible factor 1α (HIF1α) ones, leading to overexpression of genes that are involved in early hypoxic responses, such as c-MYC [21]. In addition, Gene Ontology term analysis suggested that the major STAT3 bound-regulated genes responses controlled metabolic and angiogenic processes [21]. Overall, our data suggest that STAT3 in pericytes plays a key role in the hypoxic responses in the hyperacute phase after stroke, and the upstream cytokines IL6 and IL11 may be produced by pericytes to further activate the STAT3 pathway both in an autocrine and a paracrine way, which in turn could have an important role in the early responses to hypoxia.

In our dataset, pericytes residing in the ipsilateral stroke region showed highly upregulated levels of *Ccl2* compared to the other cells in the brain. CCL2 is a chemokine with chemotactic activity for monocytes and basophils and it is produced by a wide variety of cells [64]. Previous studies have shown that *Ccl2* levels are highly increased in the brain after stroke and that silencing the *Ccl2* gene is protective in stroke models [64], promoting repair [65]. Furthermore, CCL2 deficiency has been shown to decrease macrophage infiltration to the infarct core after stroke [66]. In a transient MCAO mouse model, CCL2 inhibition resulted in a reduction of brain edema, leukocyte infiltration, and inflammation [67]. Considering the role of CCL2 in chemotaxis and BBB remodeling, our results suggest that pericytes may contribute to immune cell recruitment in the acute phase after stroke.

ADAMTS4 was among the highest and most specific genes upregulated in the ipsilateral pericytes. ADAMTS4 is a metalloproteinase with a debated function in inflammation [68–70]. On one hand, it has been reported that ADAMTS4 negatively affects the integrity of the cerebral blood vessels, increasing the risk of hemorrhagic transformation and edema after stroke [71]. On the other hand, ADAMTS4 seems to decrease inflammation after ischemic stroke by increasing the number of microglia expressing arginase-1, a marker of alternatively activated cells with anti-inflammatory functions [69]. ADAMTS4 has previously been shown to be upregulated by smooth muscle cells in cerebral arteries in response to transient MCA occlusion in rats and mice and in the ischemic brain hemisphere of patients [69, 71]. It is conceivable that pericytes, by expressing *Adamts4*, might contribute to the clearance of the injured area or to the immune cells’ phenotypic switch toward an anti-inflammatory phenotype.

Endothelin receptor subtype B (EDNRB) and subtype A mediate the effects promoted by the binding of endothelin-1 (ET-1), the most potent vasoconstrictor factor released by endothelial cells; however, these two receptors mediate opposite effects [72, 73]. EDNRA activation leads to vasoconstriction and sodium retention, resulting in higher blood pressure [73–75], while EDNRB activation leads to the release of vasodilating factors such as nitric oxide, prostacyclin-2, and endothelium-derived hyperpolarizing factor, natriuresis, diuresis, and ET-1 clearance that ultimately reduces the blood pressure [73, 74, 76]. Interestingly, a previous study showed *Ednrb* upregulation in pericytes at 24 h after ischemic stroke using a temporal occlusion of the MCA [20], suggesting that the upregulation of *Ednrb* is not dependent on the occlusion model of the MCA, but rather due to the ischemic insult itself. Pericytes that upregulate EDNRB might be more responsive to ET-1, which could regulate the vasodilation of the blood vessels around the ischemic core promoting reperfusion mechanisms, both in the permanent and the temporal occlusion models.

RDH10 is one of the enzymes responsible for the synthesis of the endothelial retinoic acid (RA) [77]. Following ischemic brain injury, exogenous RA administration has been shown to promote neuroprotection, resulting in a smaller lesion [78, 79], enhanced BBB integrity [79], lower inflammation, and improved behavioral outcomes [80]. *Rdh10* upregulation by pericytes after ischemic stroke might implicate a role for the pericytes in neuroprotection events.

STC1 is a secreted hormone with antioxidant effects [81] and it has been observed to reduce brain dysfunction after cerebral ischemia/reperfusion by decreasing BBB permeability and oxidative stress parameters [82]. Our study is the first work, to our knowledge, suggesting that STC1 is produced by pericytes in response to an ischemic injury.

Hallmark pathway analysis revealed that some upregulated pathways, such as TNFα signaling via NF-κB, hypoxia, and apoptosis, were shared between the pericytes subclusters from the ipsilateral area at 1 h and subcluster 5 at 12 and 24 h after stroke. This suggests that subcluster 5 maintains some of the initial responses of pericytes throughout the 24-h period. However, the majority of the upregulated pathways in subcluster 5 were not present at 1 h, indicating that these pericytes undergo further activation or specialization toward a more specific response, primarily related to inflammation, angiogenesis, and immune involvement. Among the upregulated Hallmark pathways, the Myc Target V1 pathway showed one of the highest fold changes and significance values. Myc is known to promote angiogenesis by inducing the expression of pro-angiogenic factors [83, 84]. Myc is also implicated in immune cell activation and inflammation-related gene expression [85]. The upregulation of the Myc Target V1 pathway suggests an increased drive for angiogenesis and vascular remodeling in response to the stroke. Additionally, it might indicate an involvement of this subcluster in an intensified inflammatory response, potentially by immune cell recruitment and the production of cytokines and chemokines.

Importantly, our results are supported by findings from a previous work, investigating cellular changes with sc-RNA seq 24 h after the ischemic insult using a transient MCAO model [20]. In one of the pericytes clusters, the authors observed the upregulation of transcripts that we also identified in this study, such as *Il11*, *Ednrb*, *Adamts4*, *Ccl2*, and *Il6*. The authors describe that the pericytes belonging to this cluster highly expressed gene sets involved in immune functions after ischemic stroke, including Oncostatin-M-induced BBB breakdown, HIF-1, and cytokine-mediated signaling pathway. Therefore, despite the difference in stroke models, certain subclusters of pericytes respond comparably 24 h after stroke.

Interestingly, in both studies, most of the transcriptional changes are associated with one specific pericyte subcluster, identified in our analysis as subcluster 5, that is only found in the ipsilateral stroke hemisphere and only detectable from 12 h after the stroke, pointing at pericytes as plastic cells where subsets of pericytes may have different functions in response to ischemic stroke.

Previously published transcriptomic studies on ischemic stroke were either performed using bulk-sequencing methods only and therefore not suitable to detect changes in gene expression from different cell types and cellular subtypes [86–88], or restricted to one timepoint after the ischemic insult [20, 62, 88, 89]. Our work extends those studies and provides specific new insights on the transcriptomic signature of pericytes in ischemic stroke, adding important knowledge on the emerging key role pericytes have at the blood/brain interface in particular as regulators of BBB integrity and inflammation after stroke, consistent with a role of pericytes as neuroinflammatory mediators at the BBB [90]. Further studies are warranted to confirm the functional implications of potential target genes in pericytes in the future and to pave the way for therapeutics modulating the pericyte response in stroke.

### Supplementary information

ESM 1 (DOCX 41 kb)

ESM 2 (XLSX 50 kb)

ESM 3 (PDF 21900 kb)

ESM 4 (PDF 589 kb)

ESM 5 (PDF 2610 kb)

ESM 6 (PDF 1442 kb)

ESM 7 (PDF 10178 kb)
