# Supplementary material for: The Transcriptional Landscape of Pericytes in Acute Ischemic Stroke
Source: Transl Stroke Res. 2023 Jun 28;15(4):714–28. doi: 10.1007/s12975-023-01169-x (PMC11226519; doi:10.1007/s12975-023-01169-x)
Supplement: Supplementary file 1 — (DOCX 41 kb) [file 12975_2023_1169_MOESM1_ESM.docx]

| Cell clusters | Markers used for identification |  |
| --- | --- | --- |
| Neuroblasts | *Dcx^+^* | [1] |
| Pericytes | *Pdgfrβ^+^, Rgs5^+^, Atp13a5^+^, Cspg4^+^, Abcc9^+^, Acta2^-^* | [2, 3] |
| Fibroblasts | *Cola1^+^, Pdgfrα^+^, Pdgfrβ^+^* | [3, 4] |
| Smooth muscle cells | *Acta2^+^, Pgbfrβ^+^* | [3, 4] |
| Oligodendrocytes | *Mog^+^* | [3] |
| Astrocytes | *Slc1a2^+^* | [5] |
| B-cells | *Cd19^+^* | [6] |
| T-cells | *Cd3e^+^* | [7] |
| Endothelial cells | *Pecam1^+^* | [3] |
| Perivascular macrophages | *Mrc1^+^* | [7] |
| Microglia | *Aif1^+^* | [3] |
| Neutrophils | *Fpr1^+^* | [8] |
| M2 macrophages | *Arg1^+^* | [9] |
| M1 macrophages | *Cd163^+^* | [9] |
| Monocytes | *Cd38^+^, Cd163^-^, Agr1^-^* | [9] |

1. Liu, X.S., et al., *Gene profiles and electrophysiology of doublecortin-expressing cells in the subventricular zone after ischemic stroke.* J Cereb Blood Flow Metab, 2009. **29**(2): p. 297-307.

2. Duan, L., et al., *PDGFRbeta Cells Rapidly Relay Inflammatory Signal from the Circulatory System to Neurons via Chemokine CCL2.* Neuron, 2018. **100**(1): p. 183-200 e8.

3. Vanlandewijck, M., et al., *A molecular atlas of cell types and zonation in the brain vasculature.* Nature, 2018. **554**(7693): p. 475-480.

4. Dorrier, C.E., et al., *CNS fibroblasts form a fibrotic scar in response to immune cell infiltration.* Nat Neurosci, 2021. **24**(2): p. 234-244.

5. Sharma, A., et al., *Divergent roles of astrocytic versus neuronal EAAT2 deficiency on cognition and overlap with aging and Alzheimer's molecular signatures.* Proc Natl Acad Sci U S A, 2019. **116**(43): p. 21800-21811.

6. Wang, K., G. Wei, and D. Liu, *CD19: a biomarker for B cell development, lymphoma diagnosis and therapy.* Exp Hematol Oncol, 2012. **1**(1): p. 36.

7. de Saint Basile, G., et al., *Severe combined immunodeficiency caused by deficiency in either the delta or the epsilon subunit of CD3.* J Clin Invest, 2004. **114**(10): p. 1512-7.

8. Leslie, J., et al., *FPR-1 is an important regulator of neutrophil recruitment and a tissue-specific driver of pulmonary fibrosis.* JCI Insight, 2020. **5**(4).

9. Jablonski, K.A., et al., *Novel Markers to Delineate Murine M1 and M2 Macrophages.* PLoS One, 2015. **10**(12): p. e0145342.
