# Supplementary material for: The Transcriptional Landscape of Pericytes in Acute Ischemic Stroke
Source: Transl Stroke Res. 2023 Jun 28;15(4):714–28. doi: 10.1007/s12975-023-01169-x (PMC11226519; doi:10.1007/s12975-023-01169-x)
Supplement: Supplementary file 3 — (PDF 21900 kb) [file 12975_2023_1169_MOESM3_ESM.pdf]

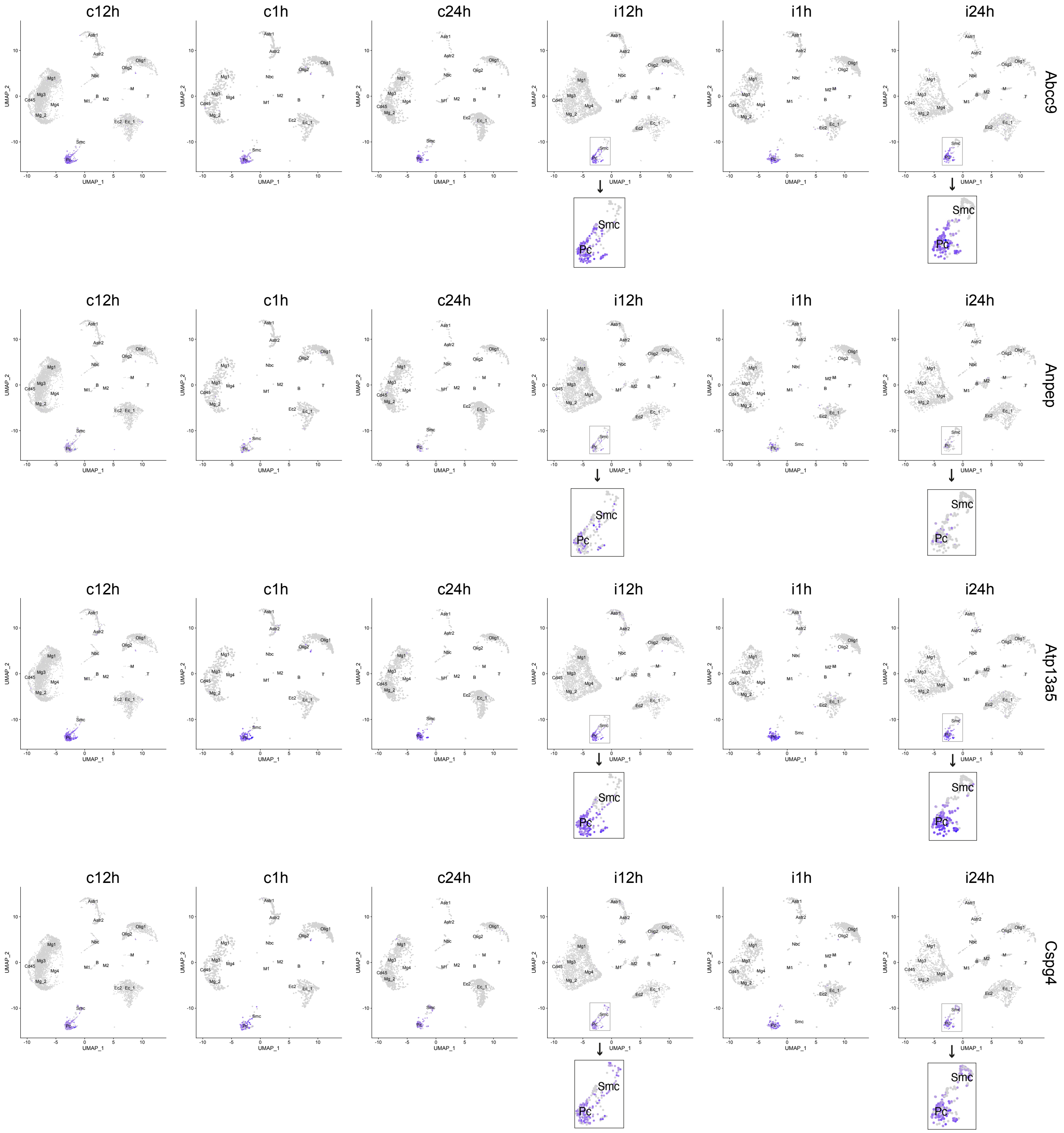

Suppl. Fig 1. Extended characterization of pericytes cluster from Figure 1. UMAP plots showing scRNA-seq data, colored by gene expression value. In addition to *Pdgfrβ* and *Rgs5*, the pericytes (Pc) express *Abbc9, Anpep, Atp13a5* and *Cspg4*. c = contralateral; i = ipsilateral; h = hour. Smc = smooth muscle cells.
