# Supplementary material for: The Transcriptional Landscape of Pericytes in Acute Ischemic Stroke
Source: Transl Stroke Res. 2023 Jun 28;15(4):714–28. doi: 10.1007/s12975-023-01169-x (PMC11226519; doi:10.1007/s12975-023-01169-x)
Supplement: Supplementary file 4 — (PDF 589 kb) [file 12975_2023_1169_MOESM4_ESM.pdf]

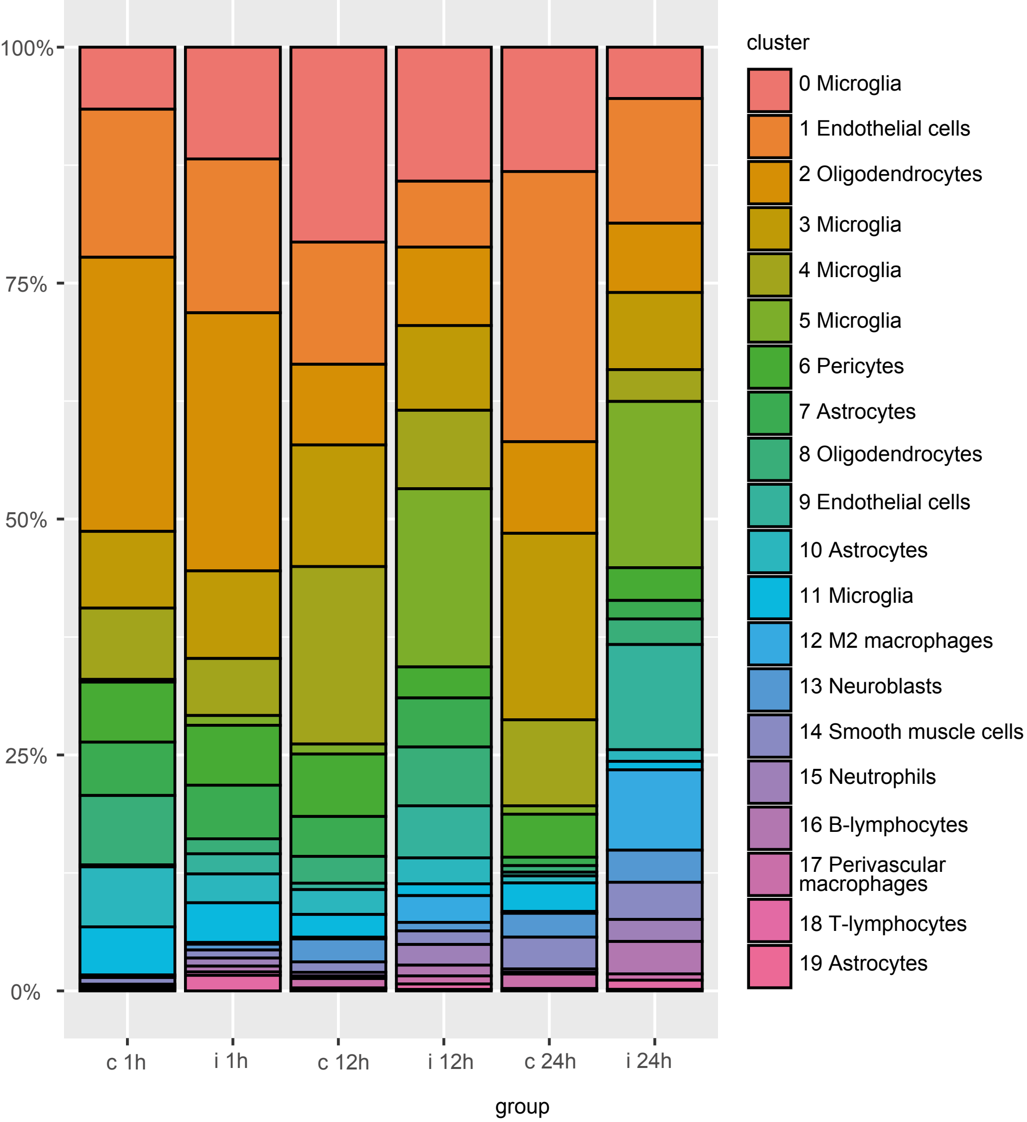

Suppl. Fig. 2 Relative distribution of cell clusters from Figure 1 across the timepoints and hemispheres. c = contralateral; i = ipsilateral; h = hour
