# Supplementary material for: The Transcriptional Landscape of Pericytes in Acute Ischemic Stroke
Source: Transl Stroke Res. 2023 Jun 28;15(4):714–28. doi: 10.1007/s12975-023-01169-x (PMC11226519; doi:10.1007/s12975-023-01169-x)
Supplement: Supplementary file 5 — (PDF 2610 kb) [file 12975_2023_1169_MOESM5_ESM.pdf]

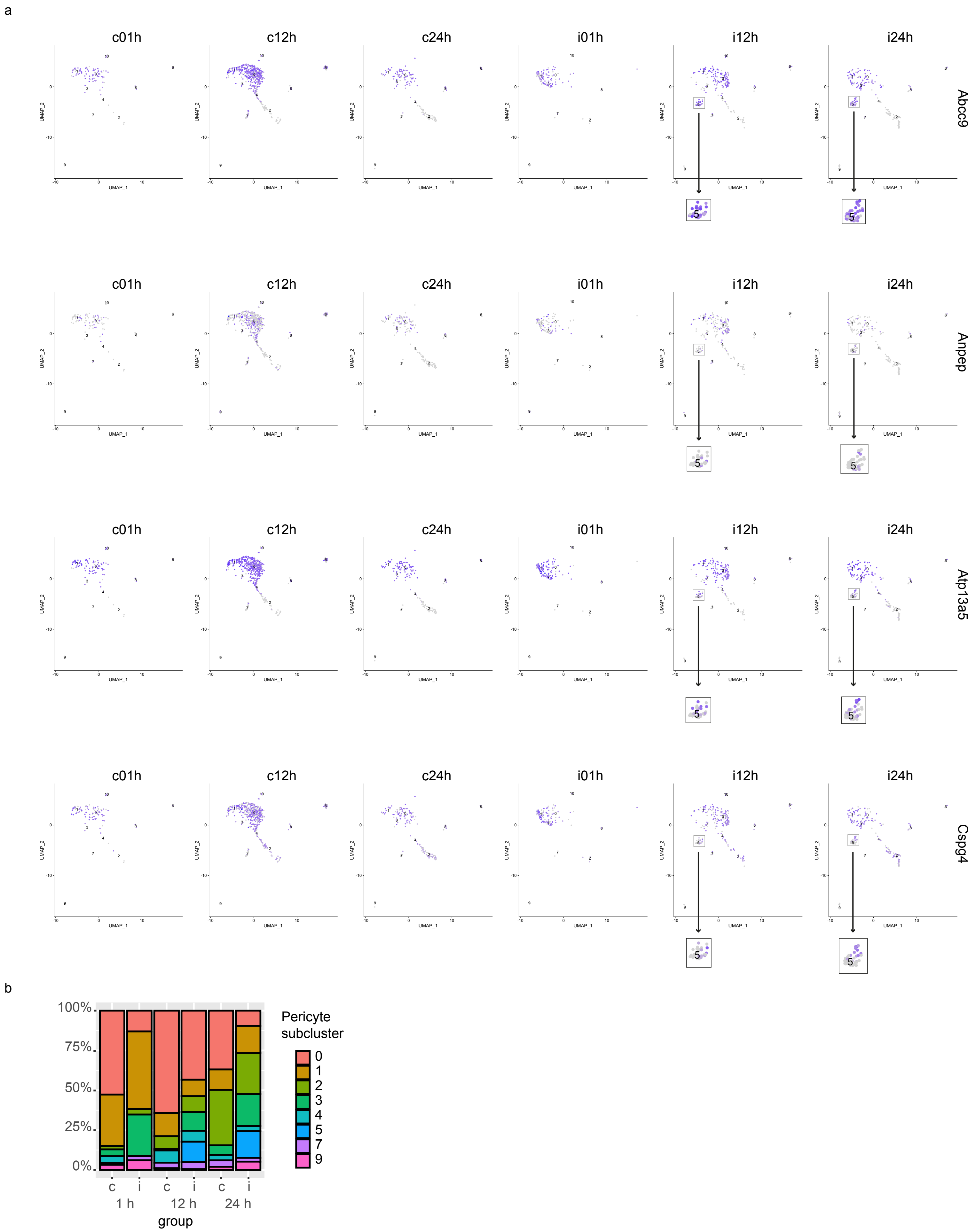


**Suppl. Fig 3.** Extended characterization of the mural cells sub-clusters from Figure 2. a) UMAP plots showing scRNA-seq data of the mural cell reclustering analysis, colored by gene expression value. The pericytes subcluster 5, in addition to *Pdgfrβ* and *Rgs5*, showes expression of *Abbc9, Atp13a5*, *Cspg4* and partially of *Anpep*. (b) Distribution of the pericytes subclusters from Figure 2 across the timepoints and hemispheres. c = contralateral; i = ipsilateral; h = hour
