# Supplementary material for: The Transcriptional Landscape of Pericytes in Acute Ischemic Stroke
Source: Transl Stroke Res. 2023 Jun 28;15(4):714–28. doi: 10.1007/s12975-023-01169-x (PMC11226519; doi:10.1007/s12975-023-01169-x)
Supplement: Supplementary file 6 — (PDF 1442 kb) [file 12975_2023_1169_MOESM6_ESM.pdf]

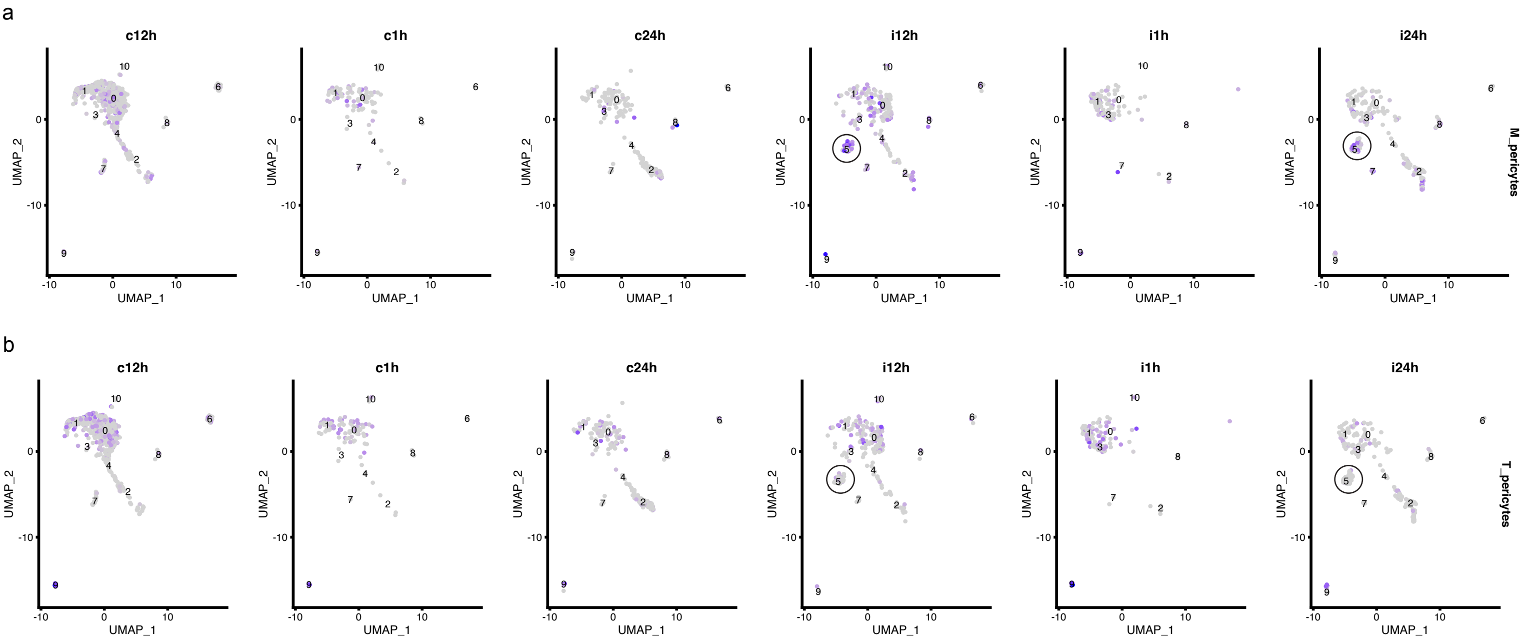

Suppl. Fig 4. UMAP plots of the mural cells subclusters showing scRNA-seq data, colored by gene expression value (a) UMAPs showing the expression of genes (*Col4a1, Col4a2, Col4a3, Col4a4, Lama4, Adamts*) associated with M- (matrix) pericytes (b) UMAPs showing the expression of genes (*Slc20a2, Slc6a1, Slc1a3, Slc12a7, Slc6a12, Slc6a13*) related to T- (transport) pericytes. c = contralateral; i = ipsilateral; h = hour. Smc = smooth muscle cells.
