# Supplementary material for: The Transcriptional Landscape of Pericytes in Acute Ischemic Stroke
Source: Transl Stroke Res. 2023 Jun 28;15(4):714–28. doi: 10.1007/s12975-023-01169-x (PMC11226519; doi:10.1007/s12975-023-01169-x)
Supplement: Supplementary file 7 — (PDF 10178 kb) [file 12975_2023_1169_MOESM7_ESM.pdf]

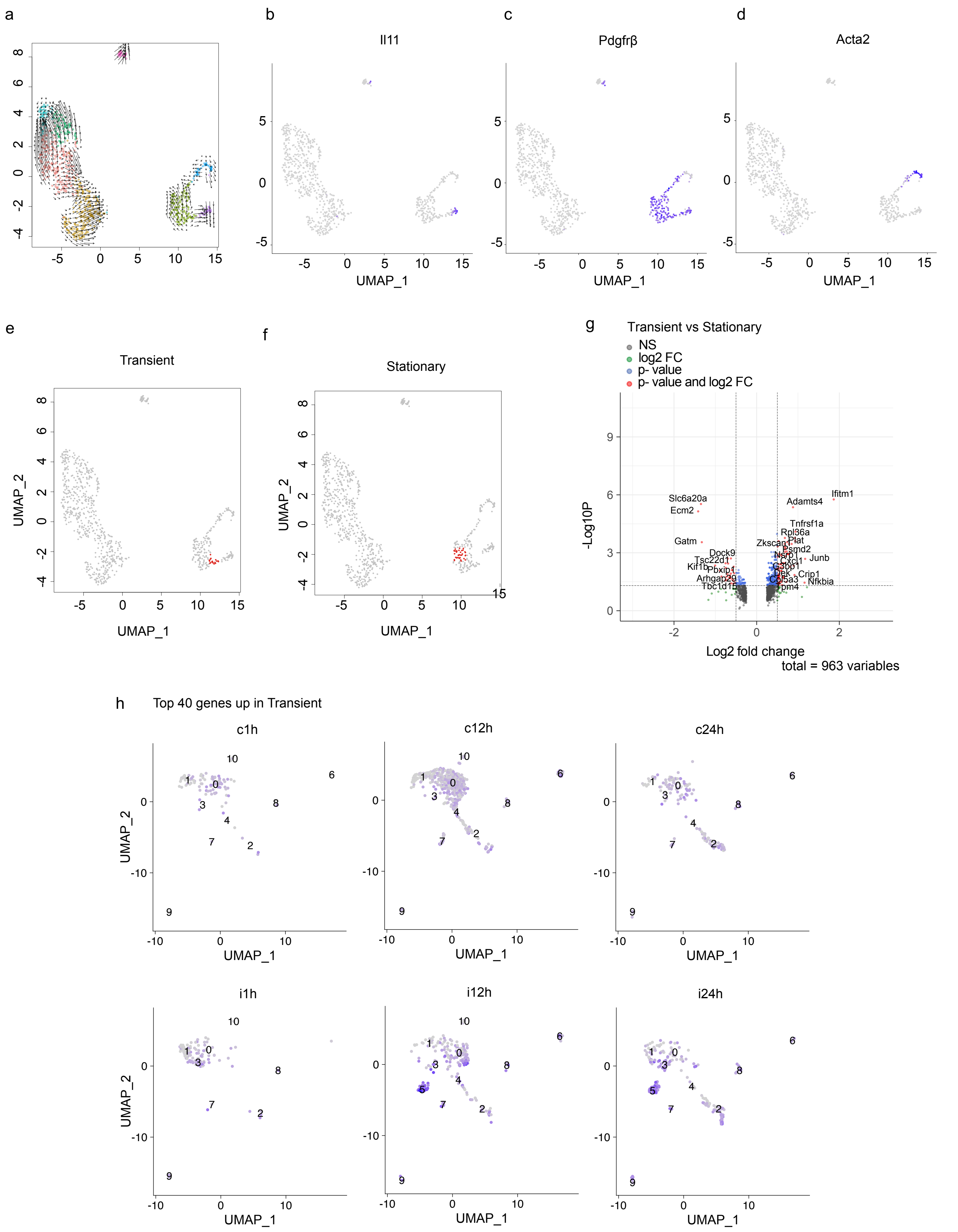

Suppl. Fig. 5 Velocyto pseudotime analysis at 12h suggests Il11-expressing cluster of pericytes as derived from pericytes (a) Velocyto of the ipsilateral 12 hours data containing mural cells and endothelial cells. Expression of *Il11* (b), *Pdgfrβ* (c) and *Acta2* (d) in the different Velocyto clusters, respectively (e) Transient and (f) Stationary pericytes towards *Il11* expressing-cells from Velocyto (g) Volcano plot showing the DEGs between the stationary and the transient pericytes (h) The top 40 upregulated DEGs were used as a gene signature and plotted in mural cell sub clusters as in Figure 2.
